# Cricket Protein Isolate Extraction: Effect of Ammonium Sulfate on Physicochemical and Functional Properties of Proteins

**DOI:** 10.3390/foods12214032

**Published:** 2023-11-05

**Authors:** Edward, Thanakorn Wongprasert, Thasorn Bunyakanchana, Panattida Siripitakpong, Kotchakorn Supabowornsathit, Tirayut Vilaivan, Inthawoot Suppavorasatit

**Affiliations:** 1 Department of Food Technology, Faculty of Science, Chulalongkorn University, Phayathai Rd., Wangmai, Pathumwan, Bangkok 10330, Thailand; edwardferdinaand@gmail.com (E.); jungga08@gmail.com (T.W.); thasorn.b@chula.ac.th (T.B.); 6470033323@student.chula.ac.th (P.S.); 2 Department of Chemistry, Faculty of Science, Chulalongkorn University, Phayathai Rd., Wangmai, Pathumwan, Bangkok 10330, Thailand; ks920920@gmail.com (K.S.); vtirayut@chula.ac.th (T.V.)

**Keywords:** cricket, insect, protein, alkaline extraction, acid precipitation, ammonium sulfate

## Abstract

Crickets are known to be a promising alternative protein source. However, a negative consumer bias and an off-flavor have become obstacles to the use of these insects in the food industry. In this study, we extracted the protein from commercial cricket powder by employing alkaline extraction–acid precipitation and including ammonium sulfate. The physicochemical and functional properties of the proteins were determined. It was found that, upon including 60% ammonium sulfate, the cricket protein isolate (CPI) had the highest protein content (~94%, *w*/*w*). The circular dichroism results indicated that a higher amount of ammonium sulfate drastically changed the secondary structure of the CPI by decreasing its α-helix content and enhancing its surface hydrophobicity. The lowest solubility of CPI was observed at pH 5. The CPI also showed better foaming properties and oil-holding capacity (OHC) compared with the cricket powder. In conclusion, adding ammonium sulfate affected the physicochemical and functional properties of the CPI, allowing it to be used as an alternative protein in protein-enriched foods and beverages.

## 1. Introduction

According to the Food and Agriculture Organization (FAO), the human population will reach nine billion by 2050 [1]. This increase will lead to an increase in food demand. In particular, fulfilling the need for protein intake has become an important issue to tackle, since protein is one of the most important nutrients for humans [2]. Thus, it is important to explore alternative protein sources in order to address this issue [3]. Nowadays, insects have become a promising alternative protein source due to their high protein content. Moreover, advantages such as lower water consumption, efficient feed conversion, and lower ammonia gas generation and emissions make the processing of insects more environmentally friendly compared with that of traditional livestock [4].

The house cricket (*Acheta domesticus*) is one of the most promising insects for use as an alternative protein source due to its high protein profile. The protein of the cricket comprises an adequate number of essential and nonessential amino acids, which can fulfill the amino acid requirements for adults and children as recommended by the World Health Organization (WHO) and FAO [5].

The use of cricket protein on the whole must overcome a large obstacle due to the negative bias of consumers regarding eating insects, since they are frequently associated with disgusting food and provide an off-flavor (soapy notes, earthy notes, and an unpleasant aftertaste) [6]. Thus, it is important to find alternative methods for incorporating crickets into foods and using them in the food industry. Altering the form of the insect to a nonvisible form such as a powder has been proven to enhance the acceptability of protein from crickets. In addition, defatting is also important, not only to tackle the off-flavor, but also to increase the extraction yield of cricket protein [6]. Moreover, many techniques could be applied to enhance the functional properties of cricket protein, such as its foaming properties, solubility, emulsifying properties, water-holding capacity, and oil-holding capacity [4]. Dion-Poulin et al. [7] reported that combining high hydrostatic pressure (HHP) with enzyme treatment could increase the solubility of cricket hydrolysates, while Zielińska [8] noted that extraction using alkaline extraction and acid precipitation could drastically improve the functional properties of cricket protein, including the water- and oil-holding capacity, foaming, and emulsification properties. In addition, Purschke et al. [9] reported that the emulsifying properties, foaming properties, and oil-holding capacity of migratory locust (*Locusta migratoria* L.) protein were improved by enzymatic hydrolysis treatment.

The protein content obtained from the extraction process could be maximized to produce protein isolate, which would have many beneficial advantages when used in the food industry. The isolation process not only increases the extraction yield but also improves the functional properties of the protein, such as its solubility, emulsifying properties, and foaming properties [10]. Alkaline extraction–acid precipitation (AEAP) is one of numerous techniques that can be employed to extract protein. This technique is used to solubilize the protein in an alkaline environment and precipitate it by altering the pH to its isoelectric point (pI) [10]. Moreover, the addition of a salt, such as ammonium sulfate, can be combined with AEAP to increase the extraction yield and purify the protein. Many studies have reported the effectiveness of AEAP not only in increasing protein content, but also in improving the functional properties of protein. A study by Gao et al. [11] reported that AEAP increased the recovery yield of yellow pea protein from 49.20% to 57.56%. Du et al. [12] reported increased solubility, water- and oil-holding capacity, foaming, and emulsifying properties of sea cucumber protein isolate after AEAP. Wang et al. [13] reported that the combination of ultrasound and AEAP positively affected the functional properties of pea protein isolate, including its solubility, water- and oil-holding capacity, foaming and emulsification capacity, stability, and gel-forming capacity. Moreover, including ammonium sulfate can promote the salting-in/out mechanisms, thus allowing higher protein content to be obtained [14]. There have been some reports on extracting the protein content of crickets using the aforementioned methods [15,16,17,18]. However, to the best of our knowledge, the effect of ammonium sulfate on the extraction yield and the physicochemical and functional properties of cricket protein isolate is largely unknown. Therefore, this study aimed to provide information regarding the effect of ammonium sulfate on the physicochemical and functional properties of the CPI.

## 2. Materials and Methods

### 2.1. Materials

House cricket (*A. domesticus*) powder (approximately 70% protein) was purchased from Protanica Co., Ltd. (Bangkok, Thailand). Sodium dihydrogen orthophosphate dihydrate (99%) and sodium phosphate dibasic dihydrate (99.5%) were purchased from Loba Chemie Pvt. Ltd. (Mumbai, India). Sodium hydroxide (NaOH) was purchased from Sigma-Aldrich (St. Louis, MO, USA). N-hexane was purchased from Macron Fine Chemical (Center Valley, PA, USA). Ammonium sulfate and hydrochloric acid (HCl) were purchased from Qrëc (Rawang, Malaysia). Sodium dodecyl sulfate (SDS), dialysis tubing, and boric acid were purchased from Thermo Fisher Scientific (Waltham, MA, USA).

### 2.2. Sample Preparation

First, we determined the protein and lipid content in cricket powder using proximate analysis following the method of Ndiritu et al. [19]. To prepare the defatted powder, cricket powder was passed through Soxhlet extraction [20] using n-hexane as the solvent. Extraction and isolation were carried out by following the methods of Jiang et al. [10]. Alkaline extraction and acid precipitation (AEAP) were conducted by dissolving 5 g of defatted cricket powder in 120 mL of 1.5% NaOH (*w*/*v*) and then shaking in a water bath at 80 °C for 1 h. Afterward, the sample was cooled to room temperature and centrifuged (6000× *g*, 10 min). After centrifugation, 1 M HCl was used to adjust the pH of the supernatant to 4.5 and precipitated parts of the sample were collected. The isolation step was conducted by adding 50 mL of ammonium sulfate at different concentrations (0%, 20%, 40%, and 60%, *w*/*v*). Subsequently, the sample mixture was kept overnight in a refrigerator (4 °C) and dialyzed overnight (buffer phosphate pH 7) by using a dialysis membrane (10 K MWCO, SnakeSkin Dialysis Tubing, Thermo Fisher Scientific, Waltham, MA, USA) before freeze-drying. The cricket protein powder samples were vacuum-sealed in aluminum foil laminated bags and stored at −18 °C for further analysis.

### 2.3. Protein Content Determination

The protein content of the sample was determined by using the Kjeldahl method (929.08) from AOAC (2002) [21], using 6.25 as the conversion factor.

### 2.4. Circular Dichroism (CD)

The secondary structure of the protein was measured via the CD technique following the method by Suppavorasatit et al. [22] with slight modification. In this process, 1 mg of protein was dissolved in 1 mL of phosphate buffer (pH 7). The CD spectra in the far UV region (190–250 mm) of each sample were measured by using a spectropolarimeter (Jasco J-815, Jasco, Japan). The sample was analyzed by using a 1 cm path length square-quartz cuvette with a Teflon cap. The percentage of secondary structure of the samples was obtained by using a web-based server, Dichroweb, which was developed by Wallace and collaborators [23].

### 2.5. Protein Solubility

The protein solubility (PS) was measured according to the method of Yong et al. [24] with some modifications. In this process, 10 mg of the sample was dispersed in 1 mL of phosphate buffer at various pH values (3, 5, 7, 9, and 11), vortexed briefly, and stored at room temperature overnight. Subsequently, the samples were centrifuged (1000× *g*, 10 min, 10 °C) and the protein content was determined by using a DC protein assay (Bio-Rad Laboratories, Hercules, CA, USA) with bovine serum albumin (BSA; Sigma-Aldrich, St. Louis, MO, USA) as protein standard. The solubility of protein was calculated according to the formula by Achouri et al. [25]:

$$PS\;\left(\%\right)=\frac{protein\;in\;supernatant}{initial\;protein}\times 100$$

### 2.6. Surface Hydrophobicity

The surface hydrophobicity was measured following the method of Zhu et al. [26] with slight modification. The freeze-dried samples were used to make protein solutions with a final concentration of 4 mg/mL by dissolving in 0.01 M phosphate buffer (pH 7) and stirring at room temperature for 2 h. Afterward, the protein was diluted serially with phosphate buffer (pH 7) to obtain concentrations of 0.5, 0.1, 0.05, 0.01, and 0.005 mg/mL. Then, 50 µL of 8-anilino-1-naphthalene sulfonate (ANS) probe (8.0 mM in 0.01 M, pH 7 buffer solution) was added to 4 mL of each protein suspension, followed by brief vortexing. Subsequently, the protein dilution was stored in the dark for 15 min. The fluorescence intensity (FI) was determined by using a fluorescence spectrophotometer (Jasco FP-6200, Jasco, Japan) at wavelengths of 365 nm (excitation) and 484 nm (emission). The FI values of buffer and protein dilution (without ANS probe) were also measured as blank samples. The net FI was obtained by subtracting the FI of the protein solution without the probe from that with the probe. The slope obtained from the plot of net FI versus protein concentration was considered as the protein surface hydrophobicity (H_0_).

### 2.7. Zeta Potential 

Zeta potential was measured by following the method of Santiago et al. [4] with modification. The protein sample (0.05 g) was dispersed in 5 mL of phosphate buffer (pH 7), and the zeta potential was measured by using a Zetasizer (Malvern Zetasizer Nano ZSP, Malvern Panalytical, Malvern, UK) using a folded capillary cell.

### 2.8. Water-Holding Capacity (WHC) and Oil-Holding Capacity (OHC)

The method of Zielińska et al. [27] with slight modification was used to measure the WHC and OHC of the samples. The WHC was measured by dissolving 0.5 g of protein sample into 20 mL of distilled water. The sample was shaken for 45 min at 250 rpm using a shaking incubator (NB-205, N-Biotek, Gyeonggi-do, Republic of Korea), then centrifuged (8000× *g*) for 15 min, and the WHC was calculated as the percentage weight of precipitate that remained after centrifugation. The OHC was measured by dispersing 0.5 g of protein sample into 10 mL of palm oil (Leela Palm Oil, Chumphon, Thailand), followed by vortexing (30 s) and centrifugation (8000× *g*, 15 min). The remaining precipitate was weighed to calculate the OHC of the sample.

### 2.9. Emulsifying Properties

Emulsifying properties, including emulsifying activity index (EAI) and emulsifying stability index (ESI), were determined following the methods of Chen et al. [28] and Meenmanee et al. [29] with modification. The protein sample (0.5 g) was dispersed in 10 mM phosphate buffer (pH 7) to a final concentration of 0.1% (*w*/*v*). Subsequently, 9 mL of protein suspension was mixed with 3 mL palm oil (Leela Palm Oil, Chumphon, Thailand) and homogenized (22,000 rpm, 5 min) to form an emulsion. Then, a 50 µL aliquot of the prepared emulsion was removed from the bottom of the test tube and diluted in 5 mL of 0.1% (*v*/*v*) sodium dodecyl sulfate (SDS) and briefly vortexed. The EAI was determined by measuring the absorbance of the dispersion at 500 nm directly while the ESI was determined by measuring the dispersion after 10 min. Both EAI and ESI were calculated by using the methods of Yin et al. [30], as shown in the equations below, where *DF*, *c*, ∅, Θ, *A*_0_, and *A*_10_ are the dilution factor, initial concentration of protein, optical path (0.01 m), fraction of oil used to form emulsion (0.25), absorbance of diluted emulsion at 0 min, and absorbance of diluted emulsion at 10 min, respectively.

$$AI\;\left(\frac{m^{2}}{g}\right)=\frac{2\times 2.303\times A_{0}\times DF}{c\times \emptyset \times \left(1-\theta \right)\times 10,000}\;ES\;\left(min\right)=\frac{A_{0}}{A_{0}-A_{10}}\times 10$$

### 2.10. Foaming Properties

Foaming capacity (FC) and foaming stability (FS) were determined by using the methods of Udomsil et al. [31] and Kunarayakul et al. [32] with slight modifications. For this process, 20 mL of distilled water was used to dissolve 0.5 g of the sample, followed by equilibration for 10 min at room temperature. The protein dispersion was then aerated using a homogenizer (22,000 rpm, 5 min). FC (%) was determined by calculating the percentage of increased volume after aeration (shown below), while FS (%) was determined as the percentage of foam remaining after 30, 60, and 90 min.

$$FC\;\left(\%\right)=\frac{volume\;after\;aeration-volume\;before\;aeration}{volume\;before\;aeration}\times 100$$

### 2.11. Statistical Analysis

A completely randomized design (CRD) was applied in all experiments. Data obtained in this study were analyzed by using one-way analysis of variance (ANOVA). Duncan’s multiple range test (DMRT) was used to determine significant differences between samples (95% confidence interval). All analyses in this study were conducted in triplicate. The data were processed using IBM SPSS Statistics software (version 29, SPSS Inc., Chicago, IL, USA).

## 3. Results and Discussion

### 3.1. Protein Content

Cricket powder as a raw material was analyzed for its protein and lipid content, which were found to be 71.2% and 10.9%, respectively. The protein content in this study was slightly higher than that in studies by Udomsil et al. [31] and Stone et al. [33], who reported a protein content of crickets of around 60–70%. This may be because the samples in the present study were from cricket powder, the production of which involved removing small parts containing less protein, such as legs and wings, before grinding and sieving. However, Stone et al. [33] reported a higher fat content, 16.1%, compared with the 10.91% found in this study. Crickets are known to be an alternative protein source with high fat content [31]. The high fat content could possibly inhibit the extraction of protein and generate an off-flavor [6].

In the present study, the defatting process was carried out before extraction using the AEAP method and isolation with ammonium sulfate added to optimize the protein content. The protein content of cricket powder, defatted cricket powder, cricket protein extracted using AEAP, and cricket protein extracted with various concentrations of ammonium sulfate (0%, 20%, 40%, and 60%, *w*/*v*) is shown in Table 1. The protein content in this study was calculated using 6.25 as Kp and reported as crude protein content. In fact, previous reports indicated that overestimation of cricket protein content could occur if a Kp value of 6.25 is used, due to the presence of nonprotein nitrogens like phospholipids, chitin, nucleic acids, and ammonia [17,34,35]. However, the recommended values (5.09 to 5.60) were not in agreement based on the total true protein from amino acid content and total nitrogen in each sample [20,35,36]. Therefore, the Kp value of 6.25 was used in the present study for comparison with other studies that used this value to calculate cricket protein content [3,19,31,33,37,38,39,40].

It was found that the protein content increased after the defatting, extraction, and isolation steps. In general, some lipids that bind to proteins may inhibit protein extraction [41]. Therefore, the defatting process would help to remove lipids and enhance the protein content after extraction [6]. The alkaline extraction–acid precipitation (AEAP) process increased the protein content from approximately 78% to 82%. This occurred due to the utilization of sodium hydroxide, which can increase protein solubility and separate protein from other components, such as fat [42]. In addition, hydrochloric acid was used to bring the protein back to its isoelectric point, where it has a net charge of 0, and thus it was precipitated, thereby increasing the protein extraction efficiency [42]. The protein content of the AEAP sample was higher compared with the cricket protein concentration reported by Ndiritu et al. [19] and the cricket protein hydrolysate by Hall et al. [3].

The combination of AEAP with the inclusion of various concentrations of ammonium sulfate showed a positive trend by enhancing the content of cricket protein (Table 1). In addition, there was no significant difference (*p* > 0.05) between AEAP cricket protein powder and that with 20% (*w*/*v*) ammonium sulfate (20 AS). However, there was a significant difference between the samples extracted with 40% and 60% ammonium sulfate (40 and 60 AS). The highest protein content of 94.0% was found in the 60 AS sample, which could be classified as cricket protein isolate (CPI) since it exceeded 90% as the minimum requirement for protein isolate classification [43]. The enhanced protein content with the inclusion of ammonium sulfate could be explained by the salting-in and salting-out mechanisms [44]. Briefly, salting-in occurs when the presence of the salt ion of ammonium sulfate (NH_4_^+^) causes an increase in protein solubility. However, a gradual addition of ammonium sulfate would cause competition between salt ions (SO_4_^2−^) and protein molecules in binding with water molecules. Ultimately, this competition could force the protein molecules to expose their hydrophobic groups, leading to aggregation and precipitation (salting-out). The increase in protein content via the inclusion of 60% ammonium sulfate in this study is in agreement with a study by Waglay et al. [14], who reported that the inclusion of 40% to 60% ammonium sulfate successfully enhanced potato protein content from 70.1% to 98.6%, as well as a study by Zhang et al. [45], who used 60% ammonium sulfate to extract potato protein and found up to 83.2% protein content. Moreover, another study by Ersus et al. [46] also mentioned the effectiveness of 85% ammonium sulfate in increasing the content of protein extracted from mallow leaf from 43.5% to 61.7%.

Based on the findings of the present study, the extraction yields of the protein samples from all treatments after freeze-drying were roughly calculated at around 16% to 24% (*w*/*w*), which could be applied to produce CPI and for use in the food industry. In addition, the high CPI value obtained in this study has not been reported elsewhere. Therefore, the physicochemical and functional properties of cricket powder, defatted cricket powder, AEAP samples, and CPI samples (inclusion of 60% ammonium sulfate) were further analyzed to determine the potential applications of CPI in the food industry.

### 3.2. Surface Hydrophobicity and Zeta Potential

Surface hydrophobicity (H_0_), an important physicochemical parameter used to determine protein characteristics, is strongly related to the functional properties of proteins, such as foaming. The H_0_ and zeta potential of cricket proteins are given in Table 2. It was found that after defatting and extracting via AEAP and AS, the H_0_ of the samples increased. According to Santiago et al. [4] and Jiang et al. [10], heat treatment and alkaline extraction can unfold proteins and thus expose their hydrophobic regions. On the other hand, including a high concentration of salt can enhance H_0_ due to the salting-out mechanism, which breaks the hydration layer on the surface and disrupts the charge balance of protein molecules. Moreover, the presence of salt ions from ammonium sulfate may promote competition with protein molecules in binding with water molecules, thus leading to aggregation of the protein and ultimately increased hydrophobicity [47].

The zeta potential reflects the compositional and structural variations in proteins, which are related to their functional properties, such as solubility [28]. External factors such as pH and ionic strength affect the zeta potential [10]. As shown in Table 2, the zeta potential of cricket powder decreased to a negative value after defatting and protein extraction. However, the zeta potential of the CPI sample (−15.30) was higher than that of the defatted (−19.00) and AEAP (−23.12) samples. Negative zeta potential values reflect the greater electrostatic repulsion and separation distance between protein molecules in a suspension [10]. According to Zhu et al. [26], the greater electrostatic repulsion of the AEAP sample was probably due to the alkaline extraction–acid precipitation method, which increases the availability of hydrophobic zones. The use of extreme acid and alkaline pH during AEAP causes unfolding of the protein structure due to the change in electrostatic repulsion accompanied by the breaking of disulfide bonds, the dissociation of subunits, and the exposure of more hydrophobic groups of protein compounds, ultimately leading to increased hydrophobicity and flexibility of protein molecules [48]. The result in this study shows that the most negative zeta potential values were observed with the AEAP technique compared with other samples. In addition, it was found that the samples with ammonium sulfate added showed fewer negative zeta potential values. This may be explained by the fact that the presence of salt ions may reduce the electrostatic repulsion between protein molecules (salting-out), as mentioned above [4].

### 3.3. Protein Solubility

**Figure 1:**
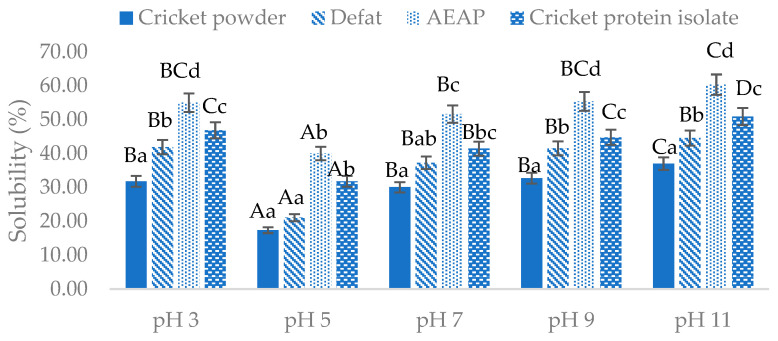
Protein solubility of cricket powder and extracted cricket protein at various pH values. AEAP, alkaline extraction–acid precipitation. Same lowercase letters for same pH indicate no significant difference (*p* > 0.05, *n* = 3); same uppercase letters for same sample across different pH values indicate no significant difference (*p* > 0.05, *n* = 3).

The protein solubility of cricket powder and extracted cricket protein at pH 3, 5, 7, 9, and 11 is presented in Figure 1. The lowest solubility of all samples was found at pH 5. This result is in agreement with a study by Zielińska et al. [27], who reported the lowest solubility was found at pH 5, while Stone et al. [33] and Ruggeri et al. [49] reported that the isoelectric point (pI) of cricket protein was around pH 4. Shifting the pH of the protein toward its isoelectric point would reduce the electrostatic repulsion between protein molecules and force the protein to aggregate and precipitate, resulting in lower solubility [50]. As shown in Figure 1, cricket protein was found to be more soluble in an alkaline than an acidic environment, as evidenced by the highest solubility for all samples being obtained at pH 11: 37.03%, 44.60%, 60.31%, and 50.94% for the cricket powder, defatted cricket powder, AEAP, and CPI samples, respectively.

Furthermore, the protein solubility results in Figure 1 are strongly related to the zeta potential results shown in Table 2. The change in the zeta potential of the cricket powder to a greater negative value occurred after the defatting, AEAP extraction, and isolating processes. This indicates greater electrostatic repulsion between protein molecules, resulting in better protein solubility [51]. Another important factor that strongly impacts solubility is ionic strength. The presence of salt ions affects the solubility of proteins, as evidenced by the high salt concentration in the CPI sample decreasing the solubility of protein [52]. This also correlates with the salting-out mechanism mentioned earlier [44].

### 3.4. Secondary Structure

Circular dichroism (CD) spectroscopy is a widely used technique for determining the secondary structures of protein samples. Table 3 displays the secondary structures of proteins in cricket powder and extracted cricket protein powder. It was found that the samples contained different α-helix, β-sheet, β-turn, and random coil structures. As shown in Table 3, random coil was the most dominant secondary structure in all samples. The reduction in α-helix occurred after the defatting, AEAP, and isolation (addition of ammonium sulfate) steps. The reduced α-helix content reflects the breaking of intermolecular hydrogen bonds, resulting in a less ordered protein structure and elevating its flexibility [53]. This finding was similar to that of a study by Mao et al. [54], who reported that alkaline extraction and acid precipitation changed the secondary structure of protein by breaking its hydrogen bonds.

As shown in Table 3, the lowest α-helix content was found in the CPI sample. This is because the addition of salt ions (ammonium sulfate) caused the rearrangement of protein molecules by exposing the buried hydrophobic region, turning them into other forms, such as β-turn and random coil [55]. According to Zhao et al. [56], α-helix and β-sheet are stabilized by intra- and intermolecular hydrogen bonds. Including a low concentration of ammonium sulfate in the form of cations (NH_4_ ^+^) increases the solubility and the α-helix content.

However, in the presence of a high concentration of anions (SO_4_^2−^), the hydrogen bonding between proteins and water molecules is weakened, leading to unfolding and stretching of the protein structure, thereby decreasing the α-helix content [56]. The exposure of the hydrophobic region of the cricket protein in this study was confirmed via measuring the surface hydrophobicity, with the CPI sample showing the highest surface hydrophobicity (Table 2). The reduction in α-helix structure due to salt ion inclusion in this study was in agreement with Sun et al. [57], who found that adding NaCl could reduce the α-helix structure of oleosin. Furthermore, Zhang et al. [47] reported that including 0.005 g/mL sodium chloride reduced the α-helix content by loosening the protein structure and destroying the secondary structures of mixed gels of wheat gluten protein and potato protein isolate.

### 3.5. Water-Holding Capacity (WHC) and Oil-Holding Capacity (OHC)

WHC is a functional property that is defined as the capability of a protein to retain water molecules [58]. Similarly, OHC is defined as the ability of a protein to retain absorbed oil [27]. Both of these functional properties are strongly determined by the composition, pH condition, and ionic strength of protein [59]. The WHC and OHC of the cricket powder and extracted cricket protein powder are shown in Table 4. A negative correlation between WHC and OHC was observed. The extraction and isolation processes reduced the WHC of the protein samples (AEAP and CPI) and positively affected the OHC, as evidenced by the elevated OHC values for the AEAP and CPI samples.

The increased WHC after defatting could be related to the increase in protein content from 71.2% to 77.6% (Table 1) and is correlated with the removal of fat content. This results in the exposure of the hydrophilic regions of protein, which can bind with water molecules [60]. On the other hand, the decreased WHC of the AEAP and CPI samples might be due to the change in the secondary structure of cricket protein after alkaline extraction–acid precipitation and the inclusion of ammonium sulfate. It can be explained that α-helix structure is mainly stabilized by hydrogen bonds between the carbonyl oxygen (-CO) and amino hydrogen (NH-) of the polypeptide chains, which can easily be destroyed under extreme acidic or alkaline conditions [61]. The results of this study agree with this theory, with a decrease in α-helix content from 39.6 to 23.4 for AEAP samples and 1.0 for CPI samples. The loss of α-helix structure leads to the weakening of hydrogen bonds, thereby reducing protein–water interactions, which was reflected in the reduced water-holding capacity of the AEAP and CPI samples [62]. Ogunwolu et al. [63] also noted that the presence of salt ions reduces WHC, since the inclusion of a large number of salt ions promotes salting-out, ultimately resulting in dehydration and reduced WHC. The WHC of cricket powder in this study was quite similar to the WHC of freeze-dried edible cricket powder (2.03 g/g) reported by Jeong et al. [64], while it was higher than the WHC of cashew nut protein isolate (1.75 g/g) reported by Liu et al. [65] and lower than that of walnut protein (5 g/g) reported by Hu et al. [66].

On the other hand, the OHC values in this study increased after defatting and extraction (Table 4). The enhanced OHC could be explained by the increased amount of protein and its exposed hydrophobic amino acids interacting and binding with the oil. Other important factors affecting OHC were the heat treatment and the extreme pH due to the use of sodium hydroxide (alkaline) and hydrochloric acid (acidic) during extraction. These can promote partial denaturation of protein, causing the exposure of more hydrophobic amino acids to interact with the oil. The OHC of CPI (3.78 g oil/g sample) was higher compared with those in previous studies on cricket protein samples by Zielińska et al. [8] and Damasceno et al. [67], in which the values were 3.10 and 2.20 g oil/g, respectively. Meanwhile, the OHC in this study was lower compared with that of the 6.7 g oil/g sample of mealworm protein hydrolysates reported by Leni et al. [50]. Furthermore, a study by Sun et al. [57] noted that including salt ions would decrease the zeta potential of protein solution and cause the exposure of more hydrophobic groups of the protein, which would enhance its ability to entrap oil.

### 3.6. Emulsifying Properties

Emulsification is defined as the ability of the protein to absorb and stabilize the oil–water interface. There are many aspects involved in the formation of a protein emulsion system, such as the movement of protein from the aqueous phase to the newly formed interface and conformational rearrangement. In general, proteins that are soluble, amphiphilic, and flexible are considered to be effective emulsifiers [68].

Table 5 gives the emulsion activity index (EAI) and emulsion stability index (ESI) of cricket powder and extracted cricket protein powder. It was found that the EAI and ESI of cricket powder increased after the defatting process. These values increased because the fat was removed, which increased the protein content. Kim et al. [69] explained that removing the fat from the protein would increase the emulsifying properties of cricket protein. Siddiq et al. [70] explained that the enhanced EAI and ESI after defatting was strongly correlated with the increased solubility of protein. The increased protein solubility was observed after defatting (Figure 1), which allowed the protein molecules to prevent the coalescence of fat by constructing a protective barrier around fat droplets, thus increasing the emulsifying properties. However, it was found that the EAI and ESI of extracted protein samples (AEAP and CPI) were decreased. Ogunwolu et al. [63] noted that increasing the protein concentration would decrease the EAI of protein, since at high concentrations, the activation energy barrier does not allow protein to migrate and it takes place in a diffusion-dependent manner.

The reduced EAI and ESI of the AEAP and CPI samples may be related to the loss of the α-helix structure of the samples. Xue et al. [71] stated that protein denaturation can occur under extreme pH during alkaline extraction–acid precipitation, which changes the secondary structures of the protein by reducing its α-helix content. Studies conducted by Guo et al. [72] and Yan et al. [73] showed that the loss of α-helix structure increased surface hydrophobicity. Excessive hydrophobicity can influence the stability of particles on the surface of oil droplets, which can affect the balance of hydrophobic and hydrophilic regions in the emulsion system. This excessive hydrophobicity might be correlated with the H_0_ of the AEAP (746.11) and CPI (956.22) samples, which were higher compared with that of the cricket powder (71.42). The EAI values in this study were higher than those in a study by Dion-Poulin et al. [7], who reported values of around 11.86–13.32 m^2^/g. Furthermore, the excessive salt ions being another cause of the exposure of hydrophobic regions of protein is in line with studies by Hu et al. [66] and Lawal et al. [74].

### 3.7. Foaming Properties

Foaming capacity (FC) is defined as the capability of protein molecules to be dissolved and unfolded rapidly in order to form a cohesive layer around a gas bubble, while foaming stability (FS) is the ability of protein molecules to form a stable foam by forming a continuous intermolecular polymer that traps air [10]. Table 6 shows the FC and FS of cricket powder and extracted cricket protein powder. Interestingly, it was found that cricket powder was unable to form a foaming system. This may be explained by the fact that the fat content can interrupt protein interactions at the air–water interface and inhibit the generation of a foaming system [58]. Thus, the defatting step is necessary to optimize the construction of a foaming system.

The results in Table 6 show that the defatted sample could form foam, but the stability of the foam was not very good. Moreover, samples from AEAP and ammonium sulfate inclusion showed higher FC and FS compared with the defatted sample. The CPI sample showed the highest FC and had the most stable foaming system. The results for foaming properties in this study were correlated with the results for protein solubility (Figure 1) and surface hydrophobicity (Table 2). This can be explained by the alkaline extraction method causing partial protein unfolding, which increases the ability of protein to undergo rapid conformational changes at the interface and rapid adsorption at the air–water interface [59]. In addition, including ammonium sulfate as salt ions may increase both FC and FS due to the exposure of hydrophilic residue and higher protein solubility during the isolation step [10]. These findings are positively related to those of a study by Yuliana et al. [75], who reported increasing FS of cashew nut shell protein from 56.03% to 76.91% at 60 min after the addition of salt, which increased the surface activity and solubility of the protein.

The FC and FS of CPI in this study were quite similar to those in a study by Jeong et al. [64], who reported values of 61.1% and 73.9% (30 min), respectively, for cricket protein concentrate. However, the CPI in this study showed remarkable foaming stability. This may be correlated with the high protein content in CPI, which was more than 90%. This may have increased the stability of the foaming system, since it exhibited faster adsorption and a smaller and more homogeneously distributed bubble size, which eventually led to a more stable foaming system [76]. Moreover, the high protein content of CPI would provide an advantage in protecting against the coalescence of bubbles due to a more stretchable interface in the foaming process [76].

## 4. Conclusions

The effect of including ammonium sulfate on the physicochemical and functional properties of cricket protein isolate (CPI) was investigated in this study. The results show that the highest protein content, up to ~94%, was obtained with the inclusion of 60% ammonium sulfate. However, the addition of high concentrations of salt ions led to decreased solubility. On the other hand, defatting, extraction, and isolation altered the secondary structure of cricket proteins by decreasing α-helix while increasing random coil. Regarding the functional properties, these techniques decrease the water-holding capacity and emulsifying properties of CPI. However, the CPI showed high oil-holding capacity and remarkable foaming stability of around 95.6–98.5%. Thus, it can be concluded that CPI is a potential food ingredient that could be implemented in the food industry in the near future.

## Figures and Tables

**Table 1:** Protein content of cricket powder and extracted cricket protein powder.

Sample	Protein Content (%)
Cricket powder	71.2 ^a^ ± 0.58
Defatted cricket powder	77.6 ^b^ ± 2.67
AEAP	82.3 ^c^ ± 1.75
20 AS	84.6 ^cd^ ± 2.02
40 AS	87.5 ^d^ ± 1.75
60 AS	94.0 ^e^ ± 2.02

AEAP, alkaline extraction–acid precipitation; 20 AS, AEAP + 20% ammonium sulfate; 40 AS, AEAP + 40% ammonium sulfate; 60 AS, AEAP + 60% ammonium sulfate. Mean ± standard deviation (*n* = 3); different letters within a column indicate significant differences (*p* ≤ 0.05).

**Table 2:** Surface hydrophobicity (H_0_) and zeta potential of cricket powder and extracted cricket protein powder.

Sample	Surface Hydrophobicity	Zeta Potential
Cricket powder	71.42 ^a^ ± 1.95	2.66 ^a^ ± 0.30
Defatted cricket powder	280.19 ^b^ ± 0.99	−19.00 ^c^ ± 0.30
AEAP	746.11 ^c^ ± 0.55	−23.12 ^d^ ± 0.35
CPI	956.22 ^d^ ± 0.14	−15.30 ^b^ ± 0.36

AEAP, alkaline extraction–acid precipitation; CPI, cricket protein isolate. Mean ± standard deviation (*n* = 3); different letters within a column indicate significant differences (*p* ≤ 0.05).

**Table 3:** Secondary structures of proteins in cricket powder and extracted cricket protein powder.

Sample	Secondary Structure (%)			
	α-Helix	β-Sheet	β-Turn	Random Coil
Cricket powder	39.6	1.8	2.9	53.2
Defatted cricket powder	20.9	24.3	19.3	40.2
AEAP	23.4	24.5	0.4	51.5
CPI	1.0	10.8	28.5	59.9

**Table 4:** Water-holding capacity (WHC) and oil-holding capacity (OHC) of cricket powder and extracted cricket protein powder.

Sample	WHC (g Water/g Sample)	OHC (g Oil/g Sample)
Cricket powder	2.01 ^c^ ± 0.01	1.6 ^a^ ± 0.00
Defatted cricket powder	2.26 ^d^ ± 0.06	1.83 ^b^ ± 0.00
AEAP	0.28 ^a^ ± 0.02	3.72 ^c^ ± 0.01
CPI	0.40 ^b^ ± 0.01	3.78 ^d^ ± 0.01

AEAP, alkaline extraction–acid precipitation; CPI, cricket protein isolate. Mean ± standard deviation (*n* = 3); different letters within a column indicate significant difference (*p* ≤ 0.05).

**Table 5:** Emulsifying properties of cricket protein powder and extracted cricket protein powder.

Sample	EAI (m^2^/g Protein)	ESI (min)
Cricket powder	54.25 ^c^ ± 0.54	22.26 ^b^ ± 0.24
Defatted cricket powder	67.45 ^d^ ± 0.23	35.43 ^c^ ± 0.23
AEAP	34.65 ^a^ ± 0.44	18.50 ^a^ ± 0.17
CPI	35.41 ^b^ ± 0.32	18.85 ^a^ ± 0.26

EAI, emulsion activity index; ESI, emulsion stability index; AEAP, alkaline extraction–acid precipitation; CPI, cricket protein isolate. Mean ± standard deviation (*n* = 3); different letters within a column indicate significant differences (*p* ≤ 0.05).

**Table 6:** Foaming properties of cricket powder and extracted cricket protein powder.

Sample	Foaming Capacity (%)	Foaming Stability (%)			
		0 min	30 min	60 min	90 min
Cricket powder	ND	ND	ND	ND	ND
Defatted cricket powder	111.67 ^a^ ± 2.89	100 ^D^	62.68 ^Cb^ ± 4.48	26.86 ^Bb^ ± 0.00	20.89 ^Ab^ ± 0.00
AEAP	166.67 ^b^ ± 7.64	100 ^D^	26.00 ^Ca^ ± 1.73	19.00 ^Ba^ ± 1.73	15.00 ^Aa^ ± 1.73
CPI	338.33 ^c^ ± 7.64	100 ^B^	98.52 ^ABc^ ± 2.26	97.04 ^ABc^ ± 2.26	95.57 ^Ac^ ± 2.26

AEAP, alkaline extraction–acid precipitation; CPI, cricket protein isolate; ND, not determined. Mean ± standard deviation (*n* = 3); different letters within a column indicate significant differences (*p* ≤ 0.05). Same lowercase letters for same time values indicate no significant difference (*p* > 0.05, *n* = 3); same uppercase letters for same sample across different time values indicate no significant difference (*p* > 0.05, *n* = 3).

## Data Availability

The data used to support the findings of this study can be made available by the corresponding author upon request.
